# Complete mitochondrial genome of *Hippophae tibetana*: insights into adaptation to high-altitude environments

**DOI:** 10.3389/fpls.2024.1449606

**Published:** 2024-08-07

**Authors:** Zhefei Zeng, Zhengyan Zhang, Norzin Tso, Shutong Zhang, Yan Chen, Qi Shu, Junru Li, Ziyi Liang, Ruoqiu Wang, Junwei Wang, La Qiong

**Affiliations:** ^1^ Key Laboratory of Biodiversity and Environment on the Qinghai-Tibetan Plateau, Ministry of Education, School of Ecology and Environment, Tibet University, Lhasa, China; ^2^ Yani Observation and Research Station for Wetland Ecosystem of the Tibet (Xizang) Autonomous Region, Tibet University, Lhasa, China; ^3^ Ministry of Education Key Laboratory for Biodiversity Science and Ecological Engineering, School of Life Sciences, Institute of Biodiversity Science, Fudan University, Shanghai, China; ^4^ Tech X Academy, Shenzhen Polytechnic University, Shenzhen, China

**Keywords:** *Hippophae tibetana*, mitochondrial genome, repetitive sequence, homologous fragments, high-altitude adaptation

## Abstract

*Hippophae tibetana*, belonging to the Elaeagnaceae family, is an endemic plant species of the Qinghai–Tibet Plateau, valued for its remarkable ecological restoration capabilities, as well as medicinal and edible properties. Despite being acknowledged as a useful species, its mitochondrial genome data and those of other species of the Elaeagnaceae family are lacking to date. In this study, we, for the first time, successfully assembled the mitochondrial genome of *H. tibetana*, which is 464,208 bp long and comprises 31 tRNA genes, 3 rRNA genes, 37 protein-coding genes, and 3 pseudogenes. Analysis of the genome revealed a high copy number of the *trnM-CAT* gene and a high prevalence of repetitive sequences, both of which likely contribute to genome rearrangement and adaptive evolution. Through nucleotide diversity and codon usage bias analyses, we identified specific genes that are crucial for adaptation to high-altitude conditions. Notably, genes such as *atp6*, *ccmB*, *nad4L*, and *nad7* exhibited signs of positive selection, indicating the presence of unique adaptive traits for survival in extreme environments. Phylogenetic analysis confirmed the close relationship between the Elaeagnaceae family and other related families, whereas intergenomic sequence transfer analysis revealed a substantial presence of homologous fragments among the mitochondrial, chloroplast, and whole genomes, which may be linked to the high-altitude adaptation mechanisms of *H. tibetana*. The findings of this study not only enrich our knowledge of *H. tibetana* molecular biology but also advance our understanding of the adaptive evolution of plants on the Qinghai–Tibet Plateau. This study provides a solid scientific foundation for the molecular breeding, conservation, and utilization of *H. tibetana* genetic resources.

## 1 Introduction

Mitochondria are essential organelles found in most eukaryotic cells, with a crucial role in cellular energy metabolism (Mackenzie and McIntosh, 1999; Bi et al., 2022; Li et al., 2023a). Unlike animal mitochondria, plant mitochondria are characterized by their large genomes, frequent structural rearrangements, and high inversion and recombination rates (Cheng et al., 2017; Wang et al., 2021). Since their origin from an initial endosymbiotic event, plant mitochondrial genomes have evolved substantially, resulting in their increased size and structural complexity compared with those in animals (Oda et al., 1992; Sloan et al., 2012; Skippington et al., 2015; Yang et al., 2021). For instance, the size of mitochondrial genomes can vary from 42 kb in *Mesostigma viride* (Oda et al., 1992) to 11.3 Mb in *Silene conica* (Sloan et al., 2012). This wide variation in genome size mainly results from the frequent recombination of repetitive sequences and the incorporation of exogenous sequences through intracellular or intercellular horizontal gene transfer (Bergthorsson et al., 2004; Møller et al., 2021; Zhou et al., 2022). These repetitive sequences are pivotal in maintaining the structure of noncoding regions in plant mitochondrial genomes, contributing to the development of multipartite structures within species (Møller et al., 2021).

Despite having large size and structural complexity, mitochondrial genomes in plants have lower mutation rates than nuclear and chloroplast genomes (Ye et al., 2017; Møller et al., 2021; Li et al., 2023a). However, the structure and gene order within these genomes vary considerably, with notable differences in gene numbers among different species (Maréchal and Brisson, 2010; Ye et al., 2017). Most functional genes in plant mitochondrial genomes are remarkably conserved, which is essential for understanding plant evolution and phylogenetics (Adams et al., 2002; Wang et al., 2021).

Because of the complexity of plant mitochondrial genomes and interference from the genomes of chloroplasts and plastids, sequencing them has traditionally been challenging (Zhou et al., 2022; Wang et al., 2024a). However, the advent of long-read sequencing technologies, such as Oxford Nanopore and PacBio, has considerably improved our ability to accurately cover and assemble these complex genomes (Lang et al., 2020; Espinosa et al., 2024; Bi et al., 2024a; Wang et al., 2024a). Moreover, advances in these technologies have enabled the complete sequencing of numerous plant mitochondrial genomes, providing valuable information for the study and use of plant molecular breeding and genetic resources (Bi et al., 2022; Han et al., 2022; Zhou et al., 2023; Li et al., 2023a, b; Xu et al., 2024; Bi et al., 2024a).

*Hippophae tibetana*, a dioecious plant species from the Elaeagnaceae family, is endemic to the Qinghai–Tibet Plateau and is among the shrubs that can survive at high altitudes globally (Qiong et al., 2017; Wang et al., 2022). *H. tibetana* mainly reproduces through seeds and cloning. Due to its excellent cloning ability and nitrogen-fixing capacity, *H. tibetana* often serves as a pioneer species in diverse ecosystems, such as floodplains and alpine meadows, and even on the bare land exposed by retreating glaciers, playing a crucial role in ecological restoration (Ruan et al., 2013; Wang et al., 2022). Moreover, plants in the *Hippophae* genus are rich in vitamin C, flavonoids, essential fatty acids, and other compounds possessing antioxidant, anti-inflammatory, and cardiovascular protective properties, making them valuable for medicinal uses, oil extraction, and as food (Ruan et al., 2013; Jain et al., 2022).

As a species with significant ecological and economic value, *H. tibetana* has garnered increasing attention. However, existing research primarily focuses on its chemical composition (Wei et al., 2022), pharmacological effects (Liu et al., 2016; Ding et al., 2022), phylogeography (Wang et al., 2010b; Jia et al., 2011), and whole genomics (Wang et al., 2022; Zhang et al., 2024), with studies on its mitochondrial genome still lacking. Currently, mitochondrial genome data for Elaeagnaceae species are still scarce in public databases, limiting our understanding of their genetic diversity and adaptive evolution. In recent years, significant progress has been made in the chromosome-level whole genome assembly of *H. tibetana*, with two chromosome-level genomes already published (Wang et al., 2022; Zhang et al., 2024). These data not only provide essential genomic foundational information but also lay the groundwork for further exploration of its adaptive evolution. Additionally, high-precision HIFI sequencing data offer the possibility of assembling its mitochondrial genome.

In this study, we present the first complete mitochondrial genome assembly and analysis of *H. tibetana*. We examined its gene composition, repetitive sequences, codon usage patterns, nucleotide diversity, and phylogenetic relationships. In addition, we explored the gene transfer between its mitochondrial and chloroplast genomes to deepen our understanding of molecular mechanisms that enable *H. tibetana* to adapt to the harsh conditions of the Qinghai–Tibet Plateau.

## 2 Materials and methods

### 2.1 Sample collection and sequencing data

We assembled the mitochondrial genome of *H. tibetana* using high-quality HiFi reads (exceeding 10 Kb in length) generated by PacBio CCS technology. These reads were obtained from samples collected by Wang et al. (2022) in Ganzi Tibetan Autonomous Prefecture, Sichuan Province, China (N 100.1056, E 29.1806). For detailed information on the collection methods, sample preservation, and initial DNA extraction procedures, please refer to Wang et al. (2022). Specific details of the sequencing data used in this study can be found in **Supplementary Table S1**.

### 2.2 Mitochondrial genome assembly and annotation

We used PMAT v1.5.4 (Bi et al., 2024b) to perform *de novo* assembly on the *H. tibetana* HiFi reads and visualized the assembly results using Bandage v0.8.1 (Wick et al., 2015). To ensure a pure mitochondrial genome assembly, we manually removed all chloroplast and nuclear genomic fragments. We used the genomes of *Hippophae rhamnoides* and *Arabidopsis thaliana* as references. For annotation, protein-coding genes (PCGs) were identified using Geseq (https://chlorobox.mpimp-golm.mpg.de/geseq.html) (Tillich et al., 2017) and IPMGA (http://www.1kmpg.cn/ipmga/). Furthermore, tRNA and rRNA annotations were performed using tRNAscan-SE v. 1.4 (Lowe and Eddy, 1997) and BLASTN v. 2.10.1 (Chen et al., 2015), respectively. Any errors found during the annotation process were corrected using Apollo v. 1.11.8 (Lee et al., 2009). The mitochondrial genome map was generated using OGDRAW v. 1.3.1 (https://chlorobox.mpimp-golm.mpg.de/OGDraw.html). Furthermore, we assembled the chloroplast genome of *H. tibetana* using the same data with Oatk v. 1.0 (https://github.com/c-zhou/oatk), and annotated the genes using Geseq (https://chlorobox.mpimp-golm.mpg.de/geseq.html) (Tillich et al., 2017). The final annotated mitochondrial and chloroplast genomes of *H. tibetana* have been uploaded to GenBank (https://www.ncbi.nlm.nih.gov/).

### 2.3 Analysis of repetitive sequences

In the mitochondrial genome of *H. tibetana*, we identified three types of repetitive sequences: simple sequence repeats (SSRs), tandem repeats, and dispersed repeats. SSRs were analyzed using the MISA online tool (https://webblast.ipk-gatersleben.de/misa/) (Beier et al., 2017), which identified repeats ranging from 1 to 6 base pairs in length. Tandem repeats were detected using Tandem Repeats Finder v. 4.09 (http://tandem.bu.edu/trf/trf.submit.options.html) (Benson, 1999), using parameters set to identify repeats longer than 6 bp and with a match score greater than 95%. Dispersed repeats were identified using BLASTN v. 2.10.1 (Chen et al., 2015), with search settings that included a word size of 7 and an E-value of 1e-5, to eliminate redundancies and further filter out tandem repeats. The distribution of all these repetitive sequences within the mitochondrial genome was visualized using Circos v. 0.69-5 (http://circos.ca/software/download/).

### 2.4 Codon usage bias analysis

We analyzed the codon usage bias in the PCGs of *H. tibetana* and *H. rhamnoides*. Initially, PCGs were extracted using Phylosuite v. 1.23 (Zhang et al., 2020). Subsequently, we calculated the relative synonymous codon usage (RSCU) values by using MEGA v. 7.0.26 (Kumar et al., 2016). To visually represent these values, bar charts were generated using the Bioinformatics Cloud Platform of Nanjing Jisihuiyuan Biotechnology Co. Ltd. (http://112.86.217.82:9919/#/).

### 2.5 Homologous fragment analysis

We conducted a homologous fragment analysis to compare the mitochondrial genome assembled in this study with its chloroplast and whole genome, respectively. Using BLASTN v. 2.10.1 (Chen et al., 2015), we identified homologous sequences based on a minimum match rate of ≥ 70% and an E-value of ≤ 1e-5. The screening length was set to ≥ 30 bp for chloroplast and mitochondrial comparisons, and ≥ 1000 bp for mitochondrial and whole genome comparisons, following the settings by Liu et al. (2023). The identified homologous sequences were visualized using Circos v. 0.69-5 (http://circos.ca/software/download/).

### 2.6 Phylogenetic analysis

We downloaded the mitochondrial genomes of 26 plant species closely related to *Hippophae* from the NCBI database to construct a phylogenetic tree, along with the newly assembled mitochondrial genome of *H. tibetana*. Conserved PCGs shared among these species were extracted using Phylosuite v. 1.23 and aligned using MAFFT v. 7.310 (Katoh and Standley, 2013). Before constructing the phylogenetic tree, we used jModeltest v. 2.1.10 (Darriba et al., 2012) to determine the optimal nucleotide substitution model, selecting the GTR+I+G model according to the Corrected Akaike Information Criterion (AICc). Subsequently, we constructed the phylogenetic tree using the maximum likelihood method with IQ-TREE v. 1.6.12 (Nguyen et al., 2015), setting the bootstrap value at 1000. The resulting tree diagram was visualized using Figtree v. 1.4.4 (http://tree.bio.ed.ac.uk/software/figtree/) (Price et al., 2010).

### 2.7 Ka/Ks ratio evaluation

To analyze synonymous and nonsynonymous substitution rates, we selected representative species from five families within the Rosales order and two species from the genus *Hippophae*. The selected species were *Ziziphus jujuba* (NC_029809), *Hemiptelea davidii* (MN061667), *Cannabis sativa* (NC_029855), *Ficus carica* (NC_077626), and *Prunus mume* (NC_065232). Homologous protein sequences between *H. tibetana* and those in the mitochondrial genomes of other species were identified using BLASTN v. 2.10.1 (Chen et al., 2015), and shared PCGs were aligned using MAFFT v. 7.310 (Katoh and Standley, 2013). Ka/Ks values, which measure the ratio of nonsynonymous to synonymous substitutions, were calculated using the MLWL model in Ka/Ks Calculator v. 2.0 (https://sourceforge.net/projects/kakscalculator2/) (Wang et al., 2010a). The results are visually represented in a boxplot created using the R package ggplot2.

### 2.8 Nucleotide diversity (Pi) analysis

Homologous gene sequences from different species were aligned using MAFFT v. 7.310 set to the auto mode. The Pi values, which measure nucleotide diversity for each gene, were calculated using DnaSP v. 5 (Librado and Rozas, 2009).

### 2.9 Comparative analysis of the mitogenome structure

For comparative analysis, we selected representative mitochondrial genomes. Dot plots, which illustrate sequence alignments, were generated using MUMmer (4.0.0beta2) (Marçais et al., 2018), with the maxmatch parameter, aligning the sequences of *H. tibetana* genome with those of the selected species’ genomes. Homologous sequences between *H. tibetana* and the selected species were identified using BLASTN v. 2.10.1. The parameters were set to a word size of 7, an E-value of 1e-5, and a length threshold of over 300 bp to construct diagrams showing multiple homologous relationships.

## 3 Results

### 3.1 Characteristics of the *H. tibetana* mitochondrial genome

**Figure 1:**
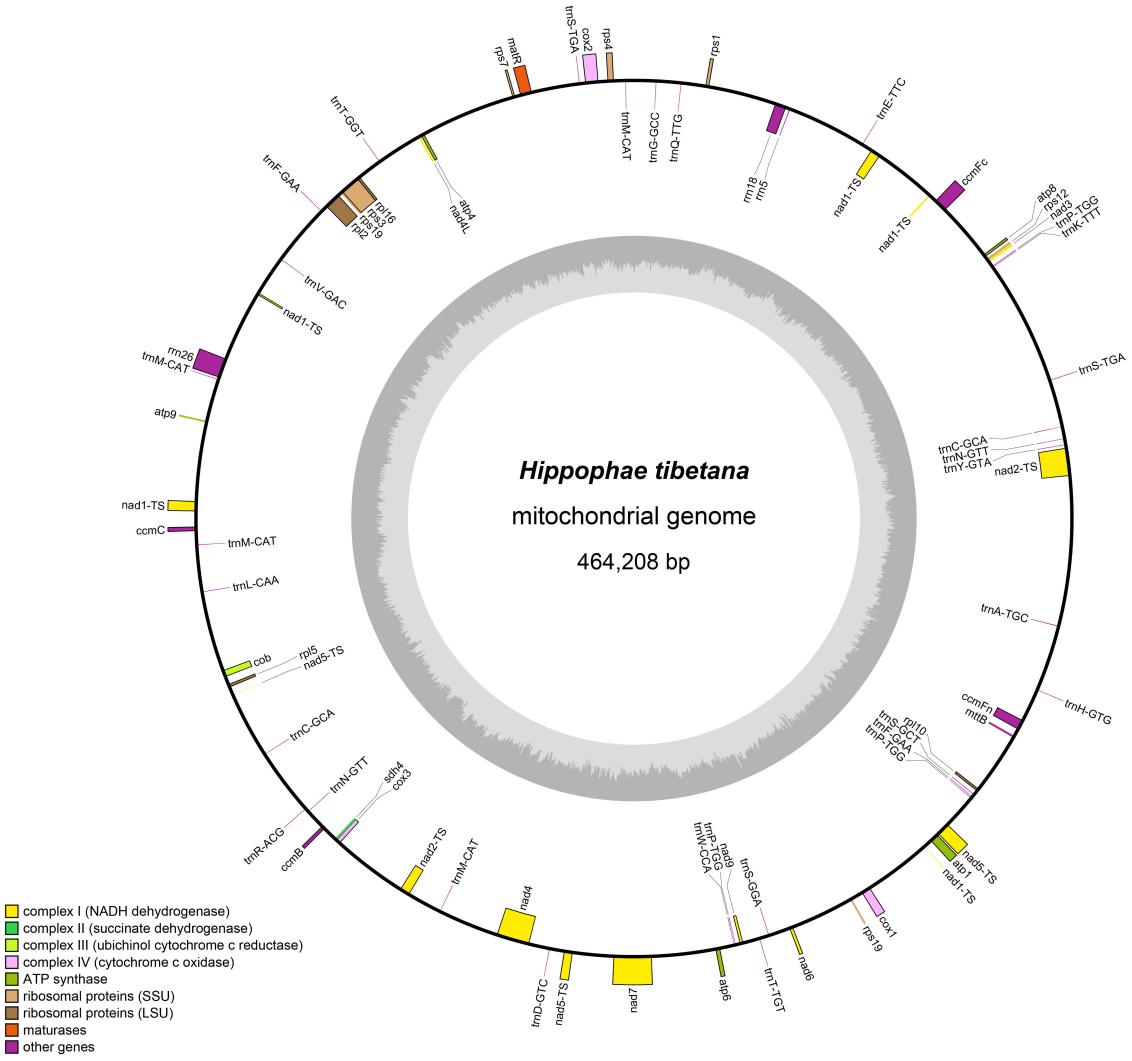
Mitochondrial genome map of *H. tibetana*. Coding genes transcribed in the forward direction are displayed on the outer rim of the circle, whereas those transcribed in the reverse direction are presented on the inner rim. The innermost gray circle indicates the GC content across the genome.

According to the draft assembly of the *H. tibetana* mitochondrial genome (**Supplementary Figure S1**), we successfully assembled the mitochondrial genome of *H. tibetana*, which has a total length of 464,208 bp and a circular structure (**Figure 1**). The genome has a GC content of 44.82%, with a bias toward AT in its base composition. Through detailed annotation, we identified 71 genes in the mitochondrial genome, encompassing 31 tRNA genes, 3 rRNA genes, 37 PCGs, and 3 pseudogenes (**Supplementary Table S2**). Of the PCGs, 25 were core mitochondrial genes, which included 5 ATP synthase genes, 9 NADH dehydrogenase genes, 4 cytochrome c biogenesis genes, 3 cytochrome c oxidase genes, 1 transporter membrane protein gene, 1 maturase gene, 1 ubiquinol cytochrome c reductase gene, and 1 succinate dehydrogenase gene. Moreover, we identified 10 noncore mitochondrial genes, of which 4 were large subunit ribosomal protein genes and 6 were small subunit ribosomal protein genes. Notably, the tRNA gene *trnA-TGC* had an intron. Of all the mitochondrial genes, eight were present in multiple copies, namely the NADH dehydrogenase gene (*nad1*), the ribosomal small subunit gene (*rps19*), and 6 tRNA genes (*trnC-GCA*, *trnF-GAA*, *trnM-CAT*, *trnN-GTT*, *trnP-TGG*, and *trnS-TGA*). Although *trnM-CAT* was found in four copies and *trnP-TGG* in three copies, the remaining multi-copy genes each had two copies.

### 3.2 Analysis of repetitive sequences

**Figure 2:**
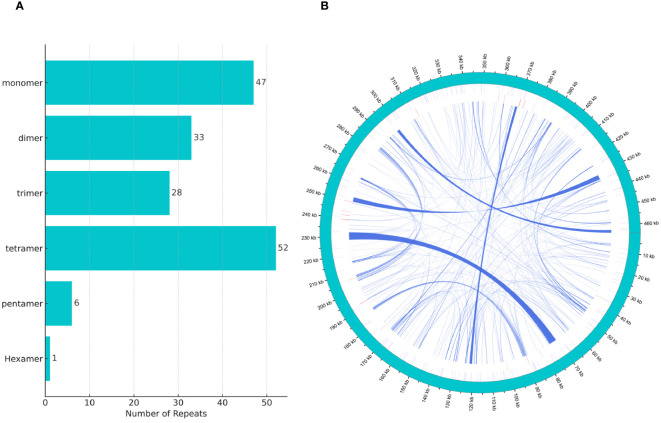
Tandem repeat distribution in the mitochondrial genome of *H. tibetana*. **(A)** Types and distribution of simple sequence repeats. **(B)** Localization of various repeat types along the genome. The outermost circle in sky blue represents the genomic scale. Simple and tandem repeats are shown in blue and red, respectively. Scattered repeats are presented in the innermost circle.

We discovered a rich array of repetitive sequences in the mitochondrial genome of *H. tibetana*, including SSRs, tandem repeats, and dispersed repeats, with a wide distribution throughout the genome, as illustrated in **Figure 2B**. SSRs are highly valued as genetic markers in species research due to their polymorphic nature, codominant inheritance, relative abundance, and broad genomic distribution (Bi et al., 2020; Zhou et al., 2023). As shown in **Figure 2A**, we identified 167 SSRs in the mitochondrial genome of *H. tibetana*. These SSR sites contained monomer, dimer, trimer, tetramer, pentamer, and hexamer repeats. Tetramer repeats were the most abundant, making up 31.14% of the total identified SSRs, followed by monomer, dimer, and trimer repeats, which constituted 28.14%, 19.76%, and 16.77% of the SSRs, respectively. Pentamer and hexamer repeats were found to be the least common. Notably, monomer repeats predominantly consisted of A/T bases, representing 93.62% of monomer SSRs, whereas dimer repeats composed of AG/CT bases accounted for 66.67% of dimer SSRs.

Tandem repeats, also known as satellite DNA, are sequences composed of core repeat units ranging from 1 to 200 bases that are repeated consecutively. These sequences are common to the genomes of both eukaryotes and some prokaryotes (Huan and Jie, 2005; Zhou et al., 2023). In the mitochondrial genome of *H. tibetana*, we identified 21 tandem repeats, with lengths varying from 9 to 39 base pairs and matching rates exceeding 72% (**Supplementary Table S3**). These results highlighted the significant diversity and potential functional importance of tandem repeats within the mitochondrial genome.

Dispersed repeats, which are scattered throughout the genome (Zhou et al., 2023), were identified extensively in the mitochondrial genome of *H. tibetana*. We found 257 dispersed repeats, with each having a length of at least 29 bp. Among these, 106 were forward repeats and 151 were palindromic repeats. Collectively, these dispersed repeats spanned 31,757 bp, accounting for 6.84% of the total mitochondrial genome. The length distribution of these repeats showed that sequences ranging from 30 to 39 bp and 40 to 49 bp were the most abundant, followed by those longer than 100 bp (**Supplementary Figure S2**). The longest forward repeat measured 2,564 bp, whereas the longest palindromic repeat reached 4,207 bp.

### 3.3 PCG codon usage analysis

We analyzed the codon usage bias in the mitochondrial genomes of *H. tibetana*, *H. rhamnoides*, and *Ziziphus jujuba*. The frequencies of amino acid codons for each species are presented in **Supplementary Figure S3** and **Supplementary Table S4**. In these species, *H. tibetana* had the highest number of codons at 10,174, followed by *Ziziphus jujuba* with 9,731 and *H. rhamnoides* with 5,808. The amino acids leucine (Leu), isoleucine (Ile), and serine (Ser) were the most frequently used across the three species, whereas cysteine (Cys) and tryptophan (Trp) were the least frequently used codons. This pattern aligns with those observed in other plant mitochondrial genomes (Dong et al., 2020; Li et al., 2023a), suggesting that the PCGs of the *H. tibetana* mitochondrial genome are relatively conserved. As shown in **Supplementary Table S2**, most PCGs in the *H. tibetana* mitochondrial genome used ATG as the start codon. However, *nad1* and *nad4L* used ACG as the start codon, possibly due to RNA editing (Ma et al., 2022). Regarding stop codons, TAA was the most commonly used, accounting for 54.29% of cases, followed by TGA at 31.43% and TAG at 14.28%.

**Figure 3:**
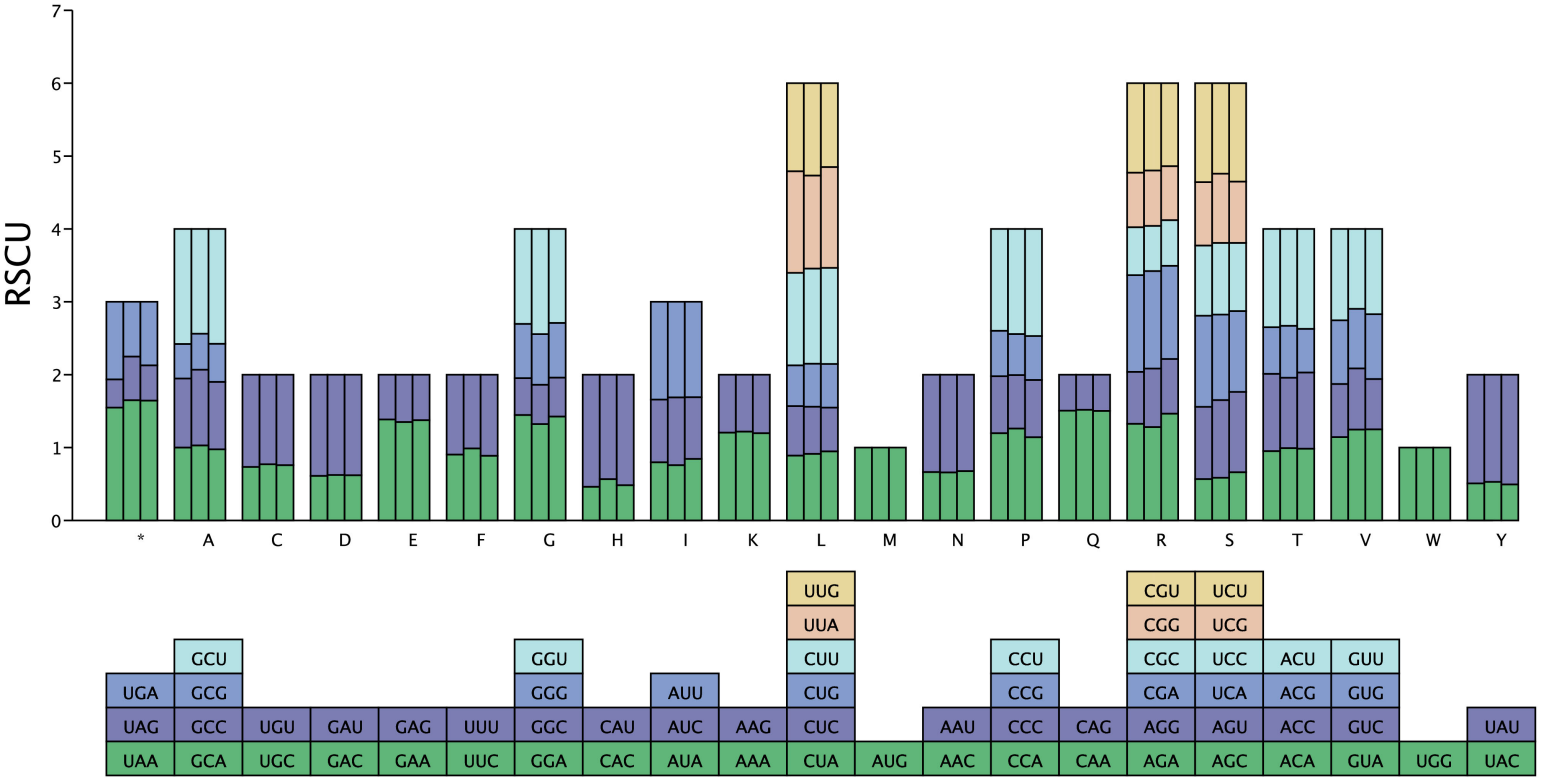
RSCU values in the mitochondrial genome of *H. tibetana*. The X axis displays different amino acids. RSCU values represent the observed frequency of each codon relative to its expected frequency under uniform synonymous codon usage.

We analyzed the codon usage bias across the three species by calculating the RSCU to determine codon preference in their mitochondrial genomes (**Supplementary Table S4**). An RSCU value of 1 indicates no codon usage bias; values < 1 suggest that a codon is used less frequently than its synonymous counterparts, whereas values > 1 indicate a higher frequency of codon usage. As shown in **Figure 3**, most mitochondrial PCGs in the three species demonstrated a preference for specific codons. In *Z. jujuba*, *H. rhamnoides*, and *H. tibetana*, the number of codons with an RSCU greater than 1 were 5,570, 3,491, and 5,938, respectively, indicating that these codons are favored over their synonymous ones. In addition, in all the three species, codons ending in A/U bases were predominantly used, comprising 93.96%, 93.47%, and 94.12% of the total, respectively. This pattern reflects a clear preference for A/U-ending codons in these mitochondrial genomes.

### 3.4 Homologous fragments

**Figure 4:**
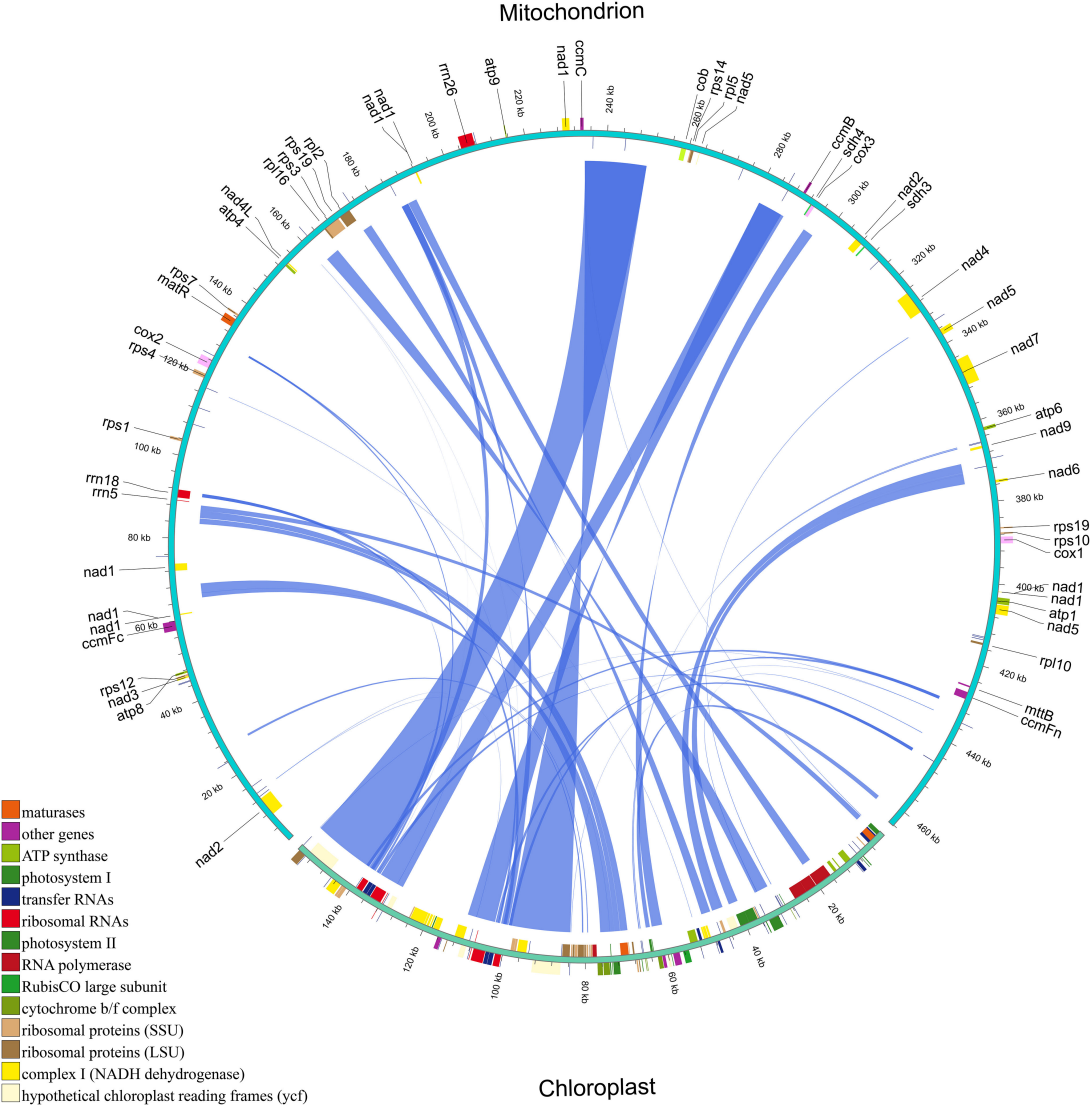
Homologous fragments between chloroplast and mitochondrial gene sequences in *H. tibetana*. Chloroplast and mitochondrial sequences are indicated, with homologous genes from the same complexes colored identically. Connecting lines in the middle represent homologous sequences.

We identified 41 homologous fragments between the mitochondrial and chloroplast genomes of *H. tibetana*, spanning a total of 83,422 bp. These homologous fragments accounted for approximately 17.97% of the mitochondrial genome (**Figure 4** and **Supplementary Table S5**), a proportion considerably higher than that observed in most other plants (Zhou et al., 2023; Li et al., 2023a; Wang et al., 2024b), suggesting active gene transfer between these organelles. The lengths of these homologous fragments ranged from 30 to 16,007 bp. We identified 19 complete chloroplast PCGs within these fragments, namely *cemA*, *ndhB*, *ndhJ*, *petA*, *petB*, *psbC*, *psbD*, *psbE*, *psbF*, *psbH*, *psbJ*, *psbL*, *psbN*, *psbT*, *rpl23*, *rps4*, *rps7*, *ycf1*, and *ycf2*. In addition, 15 tRNA genes (*trnD-GUC*, *trnF-GAA*, *trnH-GUG*, *trnI-CAU*, *trnL-CAA*, *trnL-UAA*, *trnM-CAU*, *trnN-GUU*, *trnP-UGG*, *trnR-ACG*, *trnS-GGA*, *trnT-GGU*, *trnT-UGU*, *trnV-GAC*, and *trnW-CCA*) and 4 rRNA genes (*rrn16*, *rrn23*, *rrn4.5*, and *rrn5*) were identified, along with numerous partial genes and intergenic regions. The chloroplast PCGs identified were mainly involved in photosynthesis.

In the mitochondrial and whole genomes of *H. tibetana*, we identified 583 homologous fragments ranging from 1,001 to 37,102 bp in length (**Supplementary Figure S4** and **Supplementary Table S6**). Among the 12 chromosomes of the whole genome, chromosome 2 had the highest number of homologous fragments with the mitochondrial genome, totaling 137, which is significantly more than any other chromosome (**Supplementary Figure S5**). The results (**Supplementary Table S6**) show that all genes in the mitochondrial genome have homologous counterparts in the whole genome, indicating a high frequency of gene transfer between the mitochondrial and whole genomes of *H. tibetana*.

### 3.5 Phylogenetic analyses

**Figure 5:**
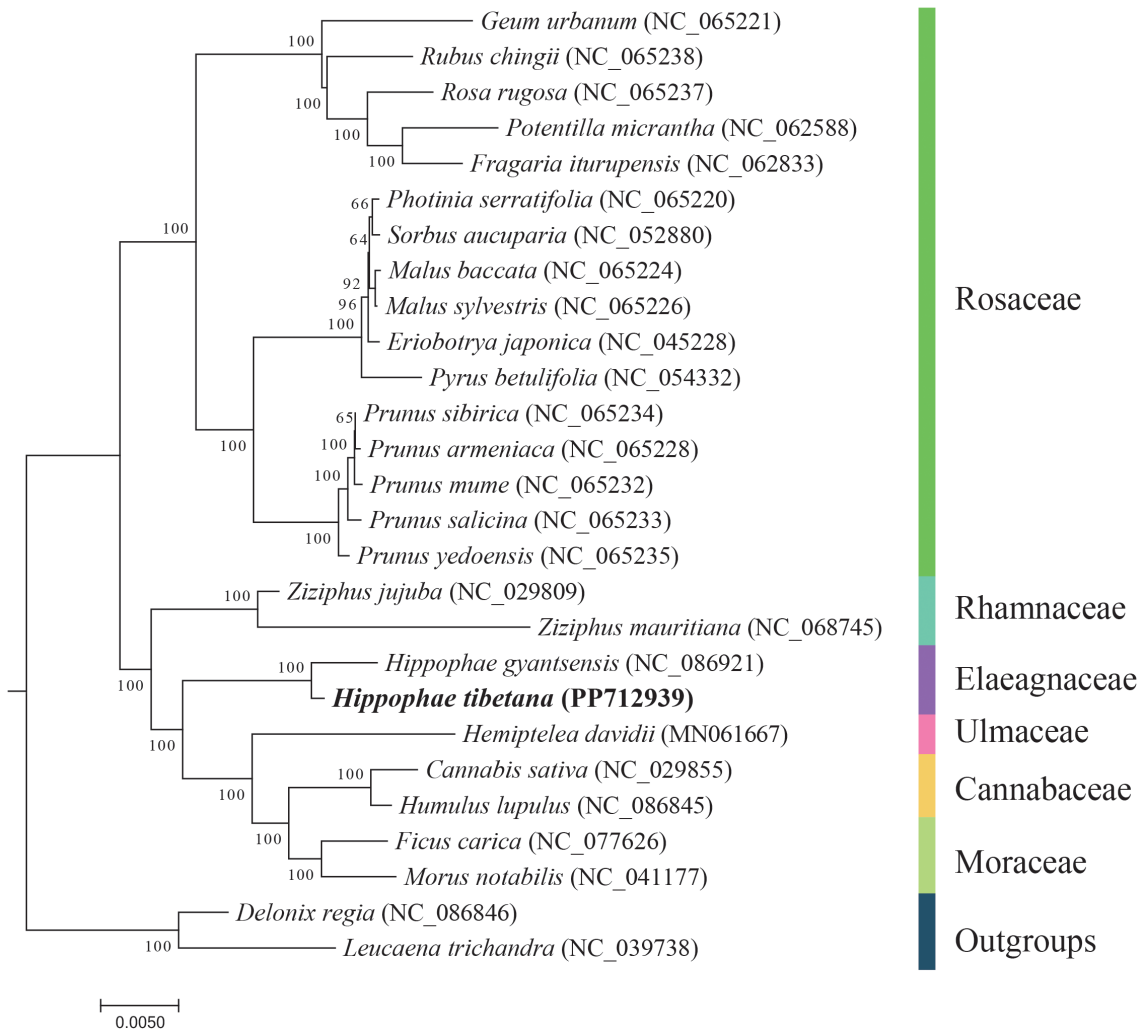
Phylogenetic tree derived from the mitochondrial protein-coding genes of 27 species. Bootstrap support values for each node are presented as percentages based on 1,000 replicates. Different families are indicated by varying colors, with *H. tibetana* highlighted in bold.

Using the DNA sequences from 13 conserved PCGs, we conducted a phylogenetic analysis involving 27 species. We selected *Leucaena trichandra* (NC_039738) and *Delonix regia* (NC_086846) from the order Fabales as outgroups. The analysis revealed that the two *Hippophae* species formed a monophyletic group, which is closely related phylogenetically to the families Rhamnaceae, Ulmaceae, Cannabaceae, and Moraceae (**Figure 5**). These findings are in alignment with the most recent classification by the Angiosperm Phylogeny Group (APG IV) (Group et al., 2016).

### 3.6 Ka/Ks ratio analysis

**Figure 6:**
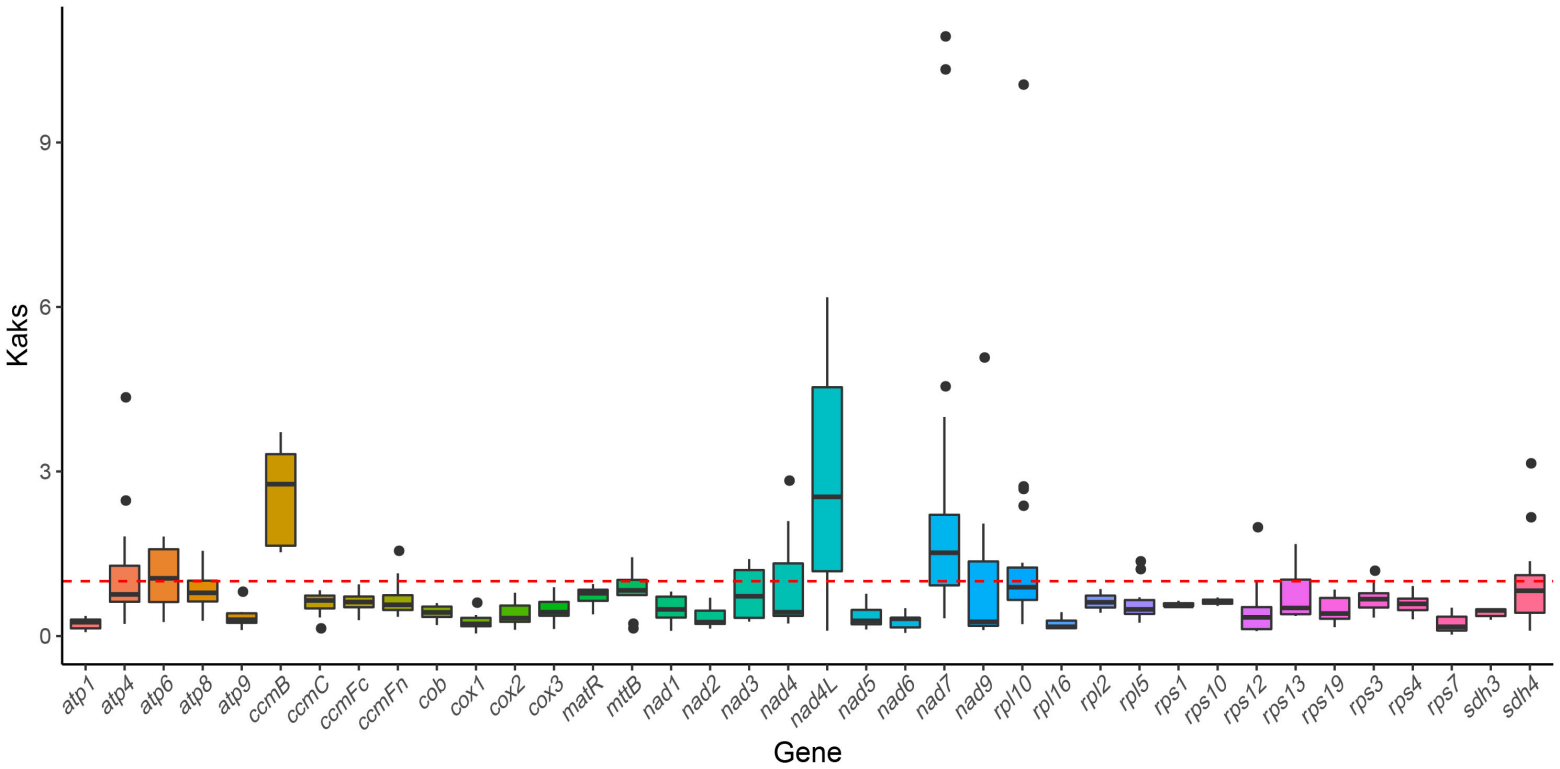
Boxplots showing pairwise Ka/Ks ratios among shared mitochondrial genes across seven rosales plants.

To examine the evolutionary selection pressures acting on mitochondrial PCGs among closely related species, we calculated the ratio of nonsynonymous (Ka) to synonymous (Ks) substitutions (Ka/Ks). A Ka/Ks ratio of 1, where Ka equals Ks, indicates neutral selection. A ratio greater than 1 (Ka/Ks > 1) suggests positive selection, indicating adaptive evolutionary changes. By contrast, a Ka/Ks ratio less than 1 indicates purifying selection (Hurst, 2002; Wang et al., 2010a), where deleterious mutations are selectively removed. In our analysis of 39 PCGs between *H. tibetana* and six other Rosales species, most genes showed signs of purifying selection (Ka/Ks < 1), indicating a predominance of conservative evolutionary pressures. However, several genes, including *atp6*, *ccmB*, *nad4L*, and *nad7*, displayed Ka/Ks ratios of > 1, which indicate their roles in specific adaptive evolutionary processes (**Figure 6**). Notably, the *atp1* gene exhibited the lowest Ka/Ks value (0.227) across all examined species, indicating strong purifying selection and high conservation during evolution.

### 3.7 Analysis of nucleotide diversity

**Figure 7:**
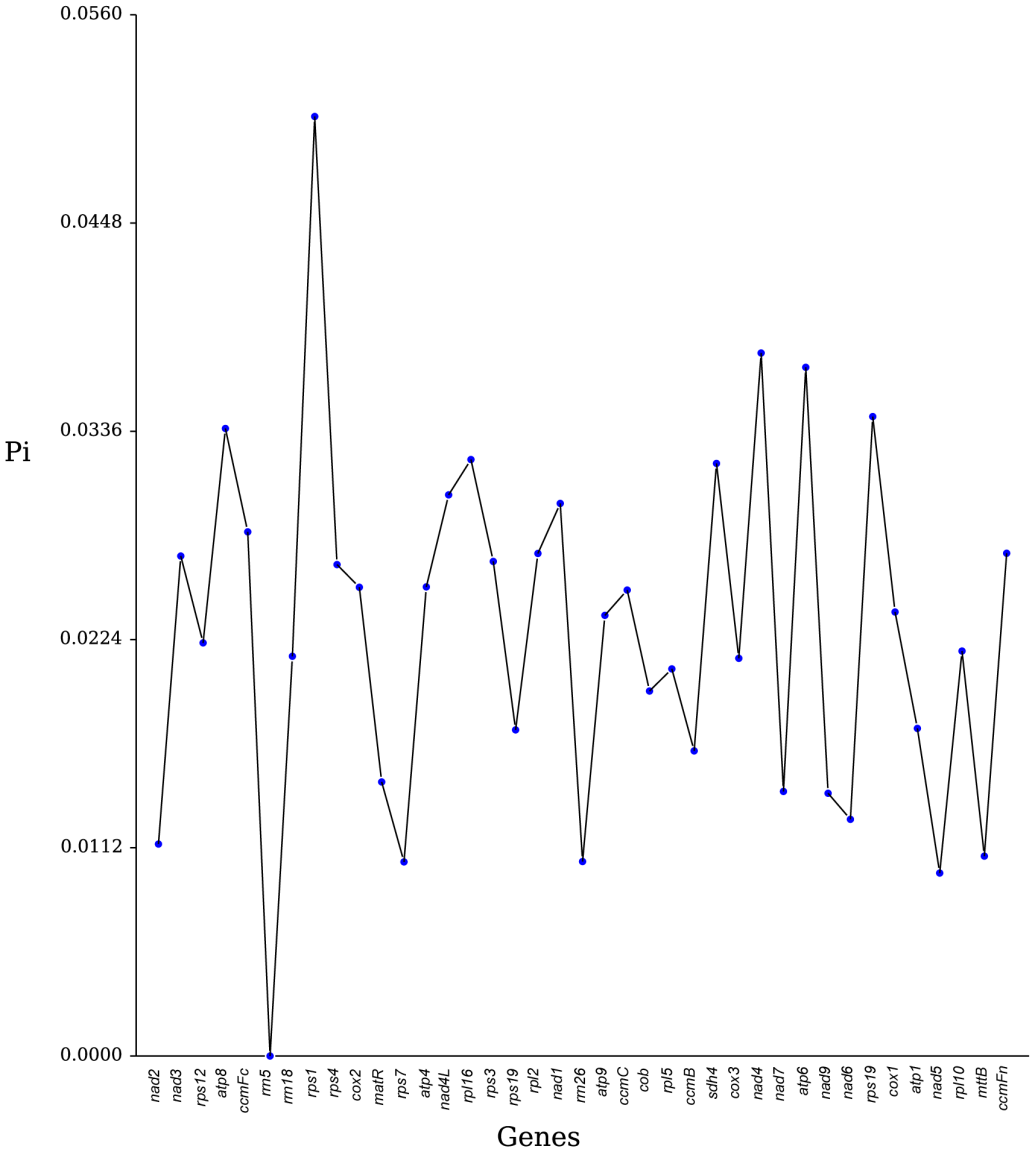
Nucleotide variability (Pi) across genes in the mitochondrial genome of *H. tibetana*.

Nucleotide diversity (Pi) is a key measure of genetic variability within species and is useful as a molecular marker in population genetics studies (Zhou et al., 2023; Li et al., 2023a). In our analysis of 36 PCGs and 3 rRNA genes from seven Rosales species, we observed that most genes exhibited low nucleotide diversity, with Pi values generally below 0.04, indicating limited genetic variation within these genes (**Figure 7**). Specifically, the genes *rps1*, *nad4*, and *atp6* demonstrated high diversity, with Pi values of 0.0505, 0.0378, and 0.0370, respectively, suggesting that these regions might have distinct evolutionary importance. By contrast, *nad5* and *nad7* were among the most conserved genes, with very low Pi values of 0.0098 and 0.0104, respectively. Among the rRNA genes, *rrn5* was extremely conserved, showing no variation (Pi = 0). Overall, these findings indicate that nucleotide diversity among the PCGs of the seven Rosales species is relatively low.

### 3.8 Collinearity analysis

**Figure 8:**
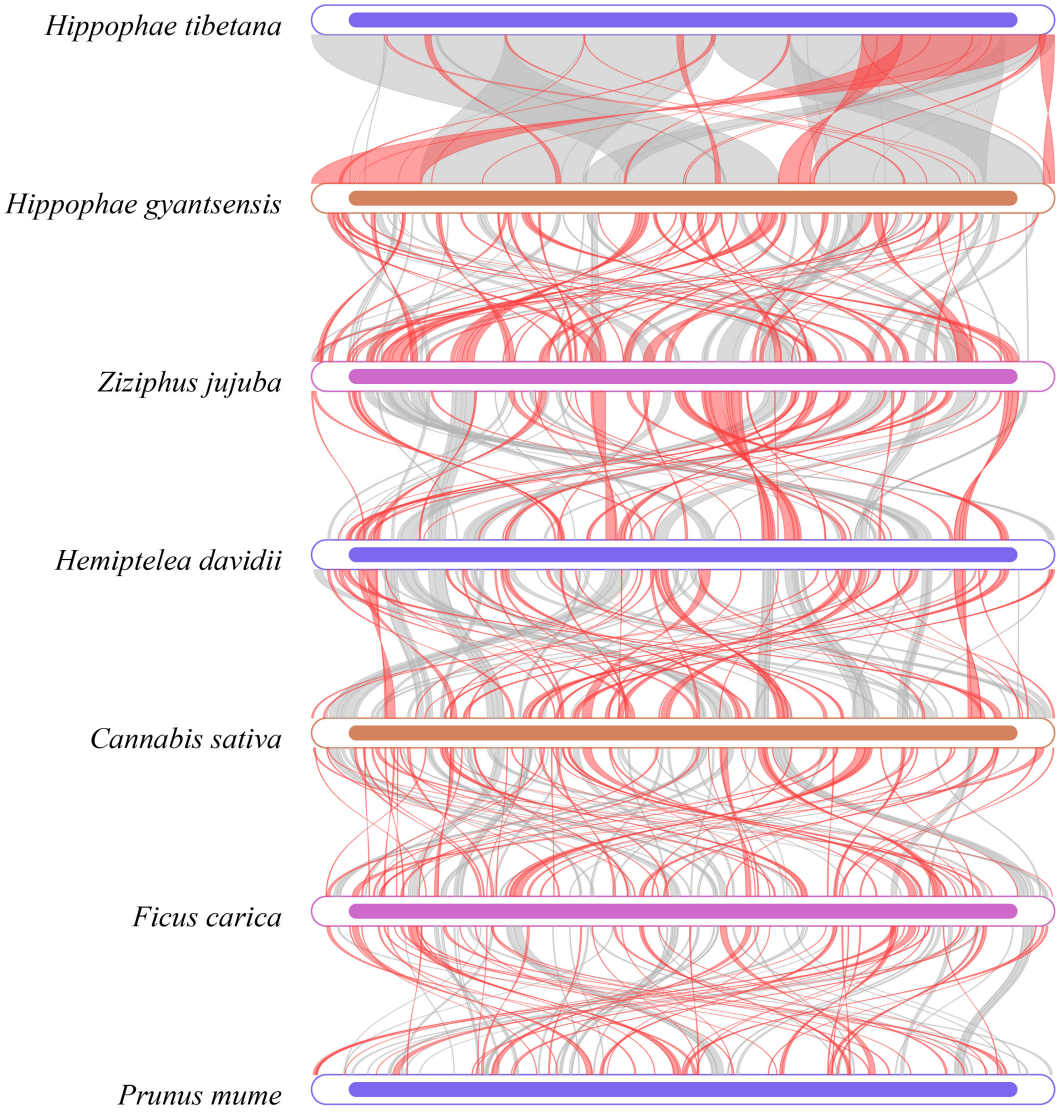
Multiple synteny plot comparing *H. tibetana* genome with those of closely related species. The boxes in each row represent a genome, and the connecting lines in the middle indicate regions of homology. The red arcs indicate inverted regions, while the gray arcs indicate regions of higher homology.

Dot plot analysis revealed long segments of highly similar (**Supplementary Figure S6**), collinear sequences between *H. tibetana* and *H. rhamnoides*, indicating strong genetic similarities. However, collinearity with other Rosales species was relatively weak. This finding suggests significant structural changes in the mitochondrial genome evolution of *Hippophae* species, though it remains relatively conserved within the same genus. Further pairwise collinearity analysis across different families within the Rosales order showed numerous homologous regions among different species, but the arrangement of these regions varied among the mitochondrial genomes (**Figure 8**). This highlights the nonconservative structural nature of the mitochondrial genomes in Rosales species, consistent with other studies showing that closely related species share the most homologous sequences (Zhou et al., 2023).

## 4 Discussion

### 4.1 *H. tibetana* mitochondrial genome rearrangement and evolution are likely driven by repeat sequences

Numerous studies have investigated the mitochondrial genome of plants because of its critical role in cellular physiology, especially ATP production and energy supply (Mackenzie and McIntosh, 1999; Bi et al., 2022; Zhou et al., 2022; Li et al., 2023a). High variability in the noncoding regions and low conservation across species make plant mitochondrial genomes a primary focus of research (Zhou et al., 2023). However, challenges in sequencing and analyzing these genome result from the accumulation of repetitive sequences, integration of chloroplast DNA, and extensive gene rearrangements (Zhou et al., 2022; Wang et al., 2024a). This study comprehensively examined the *H. tibetana* mitochondrial genome, contributing valuable data for research in genetics and evolutionary ecology. Our assembly indicated that the *H. tibetana* mitochondrial genome forms a circular structure, with a total length of 464,208 bp, a GC content of 44.82%, and a typical AT base bias. The genome contains 31 tRNA genes, 3 rRNA genes, 37 PCGs, and 3 pseudogenes. Notably, *trnM-CAT* was detected in four copies, indicating a unique evolutionary trait similar to that observed in watercress (Li et al., 2023a).

Studies have demonstrated that variations in the size of plant mitochondrial genomes are not primarily influenced by the number of coding genes or the presence of introns (Ren et al., 2023; Li et al., 2023a). Instead, these genomes often contain varying sizes of noncoding sequences, a common feature in most plant mitochondrial genomes, which contribute to size variations (Spinelli and Haigis, 2018). The analysis of repetitive sequences provides insights into the long-term genetic exchange and recombination history among species, which substantially affect the size of plant mitochondrial genomes (Møller et al., 2021). In this study of the *H. tibetana* mitochondrial genome, we discovered 167 SSRs, predominantly mononucleotide and tetranucleotide repeats. This finding suggests that variations in genome size may be largely driven by these short repetitive sequences. In addition, we identified 106 forward repeats and 151 reverse repeats but did not find any complementary or palindromic repeats. The presence of these repetitive sequences not only adds complexity to the genome but may also play a crucial role in its rearrangement and evolution.

### 4.2 Comparison of mitochondrial genomes in Rosales

With the rapid advancement of sequencing technologies, an increasing number of complete plant mitochondrial genomes have been assembled and reported, considerably enhancing comparative studies on various characteristics of these genomes (Zhou et al., 2023; Li et al., 2023a, b; Xu et al., 2024). In this study, we analyzed the mitochondrial genomes of *H. tibetana* and *H. rhamnoides* and compared them with those of other Rosales plants to deepen our understanding of differences among mitochondrial genomes of plants belonging to Rosales. Through the analyses of comparative genomic features and Ka/Ks ratios, we comprehensively explored the evolutionary dynamics of plant mitochondria (Zhou et al., 2023). Consistent with previous studies (Cheng et al., 2021; Qiao et al., 2022), most PCGs in *H. tibetana* exhibit purifying selection, suggesting a high degree of conservation in plant mitochondrial genomes. However, notable exceptions include the genes *atp6*, *ccmB*, *nad4L*, and *nad7*, which displayed signs of positive selection, and this phenomenon was observed to be more pronounced in *H. tibetana* than in other plants. This finding indicates that unique selective pressures on these genes have been the possible driving force for adaptive responses of *H. tibetana* to a high-altitude, extreme environment.

Nucleotide diversity (Pi) analysis is a valuable tool for identifying evolutionary hotspots in genomes, with Pi values indicating the degree of variation in nucleotide sequences across different plants (Li et al., 2023a). In our study, we analyzed the nucleotide diversity of 39 genes from the seven Rosales species and determined that Pi values ranged from 0 to 0.0505, with most values falling below 0.04. Notably, the PCGs *rps1*, *nad4*, and *atp6* exhibited higher variability, suggesting that these genes contain crucial evolutionary information and could serve as useful molecular markers. By contrast, *rrn5*, the most conserved rRNA gene, displayed extremely low variability. These findings provide pivotal molecular markers for future phylogenetic and population genetic studies, laying the foundation for understanding the evolutionary and genetic dynamics of plant populations.

### 4.3 Codon usage bias is related to the adaptive evolution of *H. tibetana*

Codon usage bias, which reflects variations in the frequency of synonymous codons in the coding sequences of organisms, plays a pivotal role in the transcription and translation of genetic information (Behura and Severson, 2013; Parvathy et al., 2022). This variability in codon usage among organisms is believed to result from long-term evolutionary selection within cells (Zhou et al., 2023). In this study, we observed that in *H. tibetana*, most PCGs employ typical AT start codons, and the amino acid composition in this plant is similar to those found in other angiosperms (Zhou et al., 2022; Li et al., 2023a). We examined codon usage in the mitochondrial genomes of *H. tibetana*, *H. rhamnoides*, and *Z. jujuba* and found that leucine, isoleucine, and serine were the most frequently used amino acids. This finding aligns with those from other plant mitochondrial genomes (Dong et al., 2020; Li et al., 2023a). Furthermore, our study revealed that codons with RSCU values greater than 1.00, predominantly ending with A/U bases, constituted 93.955%, 93.47%, and 94.12% of the total in *Z. jujuba*, *H. rhamnoides*, and *H. tibetana*, respectively, indicating a preference for A/U-ending codons. This preference is typical in plant mitochondria (Qiao et al., 2022) and reflects specific evolutionary and functional adaptations. Understanding codon usage bias provides insights into both the complexity of gene expression regulation and molecular mechanisms driving evolutionary selection. This study demonstrated the relationship between codon usage bias and plant adaptability, providing essential clues for understanding genetic variations among species.

### 4.4 Intergenomic sequence transfer likely contributes to high-altitude adaptation in *H. tibetana*

With the enhancement of genomic databases and use of graphical genome mapping tools, such as CGView, we can now more comprehensively analyze collinearity and DNA rearrangement events in plant mitochondrial genomes (Stothard et al., 2019). In this study, we compared the mitochondrial genomes of *H. tibetana* with those of closely related Rosales species and identified substantial structural variations. Although some collinearity exists between *H. tibetana* and *H. rhamnoides*, both species exhibit a high degree of structural nonconservation, suggesting that the *Hippophae* mitochondrial genomes have undergone frequent rearrangements throughout their evolutionary history. Moreover, our analysis revealed extensive homologous fragments across different species, indicating that mitochondrial genomes in various families of the Rosales order tend to be highly structurally diverse.

The evolutionary characteristics of mitochondrial genomes are complex, primarily manifested in structural rearrangements and gene transfer events (Cheng et al., 2017; Møller et al., 2021; Wang et al., 2021). Gene transfer between plant mitochondrial genomes and both chloroplast and nuclear genomes is a key feature of this evolutionary process, helping us understand the dynamic evolution of organellar and whole genomes (Bergthorsson et al., 2003; Rice et al., 2013; Zhou et al., 2023). In this study, we identified 41 chloroplast genome fragments in the mitochondrial genome of *H. tibetana*, totaling 83,401 bp and accounting for 17.97% of the mitochondrial genome—a proportion significantly higher than that typically observed in other plants (Li et al., 2023a; Zhou et al., 2023; Wang et al., 2024b). Notably, in *H. tibetana*, the transfer of both tRNA genes and PCGs occurs more frequently than in other plants, possibly due to the presence of longer transfer fragments. We classified 19 of these transferred PCGs and found that most of them are involved in photosynthesis, suggesting that this may be the result of *H. tibetana* adapting to high-altitude extreme environments.

In this study, we identified 583 homologous fragments between the mitochondrial genome and the whole genome of *H. tibetana*, ranging from 1,001 to 37,102 bp in length. The results showed that, among the 12 chromosomes of the whole genome, chromosome 2 contained the highest number of homologous fragments with the mitochondrial genome, totaling 137, significantly more than any other chromosome. This may be related to chromosome 2 being a sex chromosome with many repetitive sequences (Wang et al., 2022). Additionally, all genes in the mitochondrial genome have homologous counterparts in the whole genome, indicating a high frequency of gene transfer between the mitochondrial and whole genomes of *H. tibetana*.

These findings indicate that the nonconservative structure and frequent gene transfers in the *H. tibetana* mitochondrial genome may be crucial evolutionary mechanisms facilitating its adaptation to high-altitude environments. The findings of this study enhance our understanding of the structural and functional evolution of plant mitochondrial genomes. In addition, they provide valuable molecular markers and data resources for future phylogenetic and population genetic studies.

### 4.5 Phylogenetic analysis provides evidence for a close relationship of Elaeagnaceae family with other families classified under Rosales

The mitochondrial genome has become a critical tool in taxonomy, systematics, evolutionary biology, population genetics, and comparative genomics (Galtier et al., 2009; Møller et al., 2021) because of its maternal inheritance, rapid evolutionary rate, low recombination rate, and molecular marker abundance. Although short sequences have often been employed to construct phylogenetic trees (Qiong et al., 2017), this approach may not reflect phylogenetic relationships accurately because of the effect of horizontal gene transfer and variations in genetic evolutionary rates among populations. To address this issue, we constructed a phylogenetic tree by using mitochondrial PCGs from *H. tibetana* and related species, employing the maximum likelihood method. This analysis revealed a close phylogenetic relationship of the Elaeagnaceae family with the Rhamnaceae, Ulmaceae, Cannabaceae, and Moraceae families. Furthermore, the phylogenetic tree topology based on mitochondrial DNA aligns with the latest classification results from the APG IV, validating the consistency between traditional taxonomy and molecular evidence and highlighting the value of mitochondrial genome data in understanding plant phylogenetics.

## 5 Conclusions

This study successfully assembled and analyzed the complete mitochondrial genome of *H. tibetana*, revealing its distinctive genomic characteristics and the potential mechanisms underlying its adaptation to high-altitude, extreme environments. The *H. tibetana* mitochondrial genome spans 464,208 bp and contains 31 tRNA genes, 3 rRNA genes, 37 PCGs, and 3 pseudogenes, exhibiting a notable AT bias. The high copy number of the tRNA gene *trnM-CAT* and the abundance of repetitive sequences increase the complexity of the genome, potentially affecting the genome rearrangement and evolutionary processes. By analyzing nucleotide diversity and codon usage bias, we identified specific genes that facilitate the adaptation of *H. tibetana* to high-altitude conditions. Comparative genomic analysis with other Rosales plants revealed that *H. tibetana* has experienced considerable evolutionary selection pressure, particularly evident in positive selection pressure of genes such as *atp6*, *ccmB*, *nad4L*, and *nad7*. Moreover, phylogenetic analysis validated the close relationship between the Elaeagnaceae family and other related families, reinforcing the alignment between traditional taxonomy and molecular evidence. Intergenomic sequence transfer analysis revealed a substantial presence of homologous fragments among the mitochondrial, chloroplast, and whole genomes, indicating frequent sequence transfers between them, which may be linked to the high-altitude adaptation mechanisms of *H. tibetana*.

This study comprehensively analyzed the *H. tibetana* mitochondrial genome, shedding light on its genomic adaptations to extreme high-altitude environments. We identified key genes that may facilitate these adaptations, laying the foundation for future research to delve deeper into their specific roles through functional gene analysis and phenotypic validation. Such investigations will enhance our understanding of plant survival strategies under extreme conditions. The findings of this study not only furnish valuable genomic data and fresh perspectives for examining adaptive evolution in plants on the Qinghai–Tibet Plateau but also provide useful information to enhance the practice of molecular breeding and conserve *H. tibetana* genetic resources.

## Data availability statement

The original contributions presented in the study are included in the article/**Supplementary Material**. Further inquiries can be directed to the corresponding authors.

## Author contributions

ZZe: Data curation, Formal analysis, Funding acquisition, Investigation, Methodology, Software, Visualization, Writing – original draft, Writing – review & editing. ZZh: Formal analysis, Methodology, Software, Writing – review & editing. NT: Funding acquisition, Methodology, Software, Writing – review & editing. SZ: Methodology, Writing – review & editing. YC: Methodology, Writing – review & editing. QS: Methodology, Writing – review & editing. JL: Methodology, Resources, Writing – review & editing. ZL: Funding acquisition, Methodology, Resources, Writing – review & editing. RW: Methodology, Resources, Writing – review & editing. JW: Conceptualization, Data curation, Formal analysis, Funding acquisition, Methodology, Project administration, Supervision, Visualization, Writing – original draft, Writing – review & editing. LQ: Conceptualization, Data curation, Formal Analysis, Funding acquisition, Methodology, Project administration, Supervision, Visualization, Writing – original draft, Writing – review & editing.
