# Supplementary material for: Complete mitochondrial genome of Hippophae tibetana: insights into adaptation to high-altitude environments
Source: Front Plant Sci. 2024 Aug 7;15:1449606. doi: 10.3389/fpls.2024.1449606 (PMC11335646; doi:10.3389/fpls.2024.1449606)
Supplement: Supplementary file 1 [file Presentation_1.zip › Supplementary Material Figures.docx]

Supplementary Material

# **Complete mitochondrial genome of *Hippophae tibetana*: Insights into adaptation to high-altitude environments**

**Zhefei Zeng^1,2^, Zhengyan Zhang^3^, Norzin Tso^1^, Shutong Zhang^1^, Yan Cheng^1^, Qi Shu^1^, Junru Li^1^, Ziyi Liang^1^, Ruoqiu Wang^4^, Junwei Wang^1,2*^, La Qiong^1,2*^**

^1^ Key Laboratory of Biodiversity and Environment on the Qinghai-Tibetan Plateau, Ministry of Education, School of Ecology and Environment, Tibet University, Lhasa, China

^2^ Yani Observation and Research Station for Wetland Ecosystem of the Tibet (Xizang) Autonomous Region, Tibet University, Lhasa, China

^3^ Ministry of Education Key Laboratory for Biodiversity Science and Ecological Engineering, School of Life Sciences, Institute of Biodiversity Science, Fudan University, Shanghai, China

^4^ Shenzhen Polytechnic University, Shenzhen, China

*** Correspondence:**

Corresponding Author, Junwei Wang [(jwyx12240315@126.com)](mailto:(jwyx12240315@126.com)) and La Qiong [(lhagchong@163.com](mailto:(lhagchong@163.com))

# Supplementary Figures


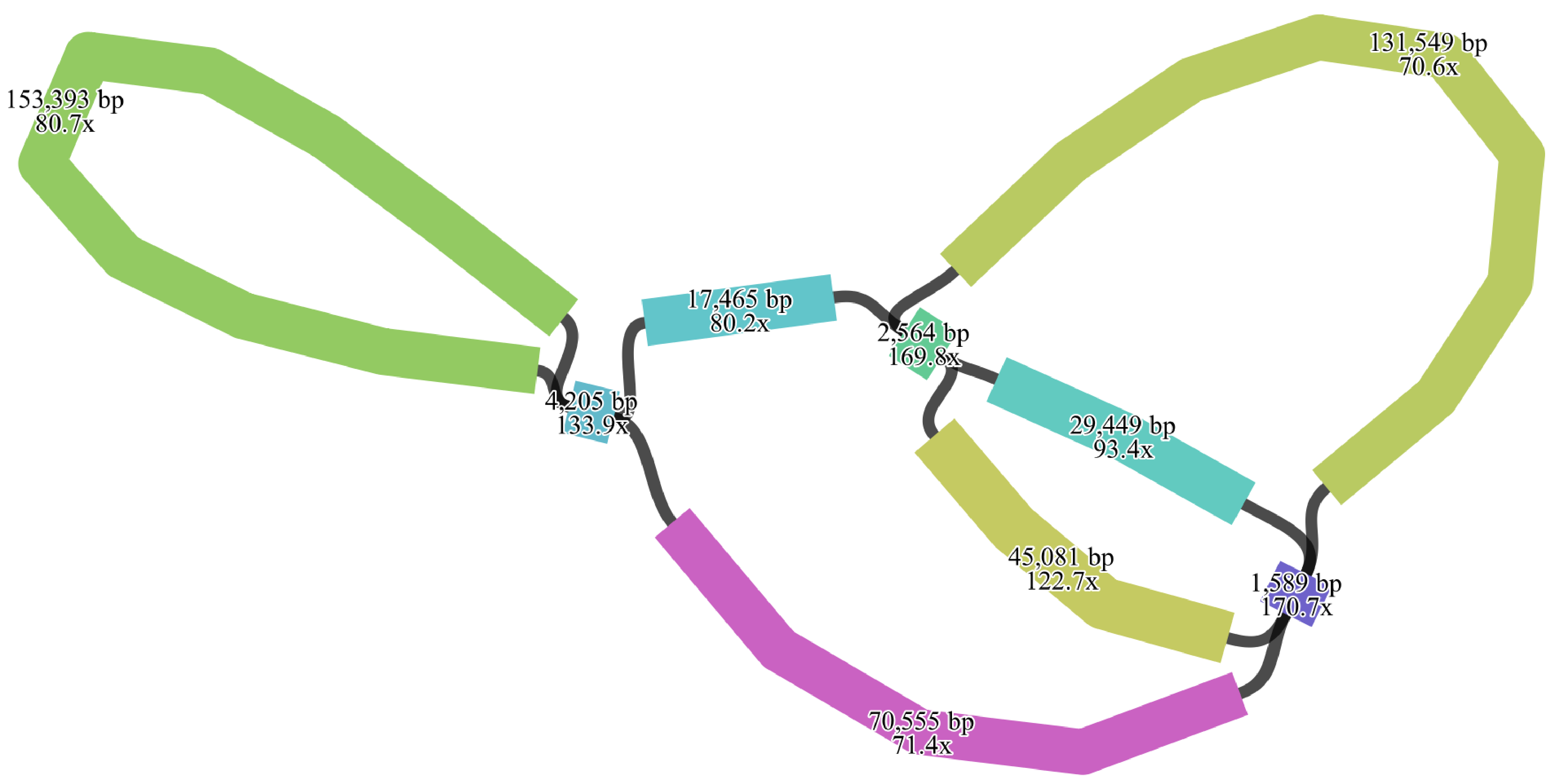


**Supplementary Figure 1.** The assembly result of the mitochondrial genome of *H. tibetana.*


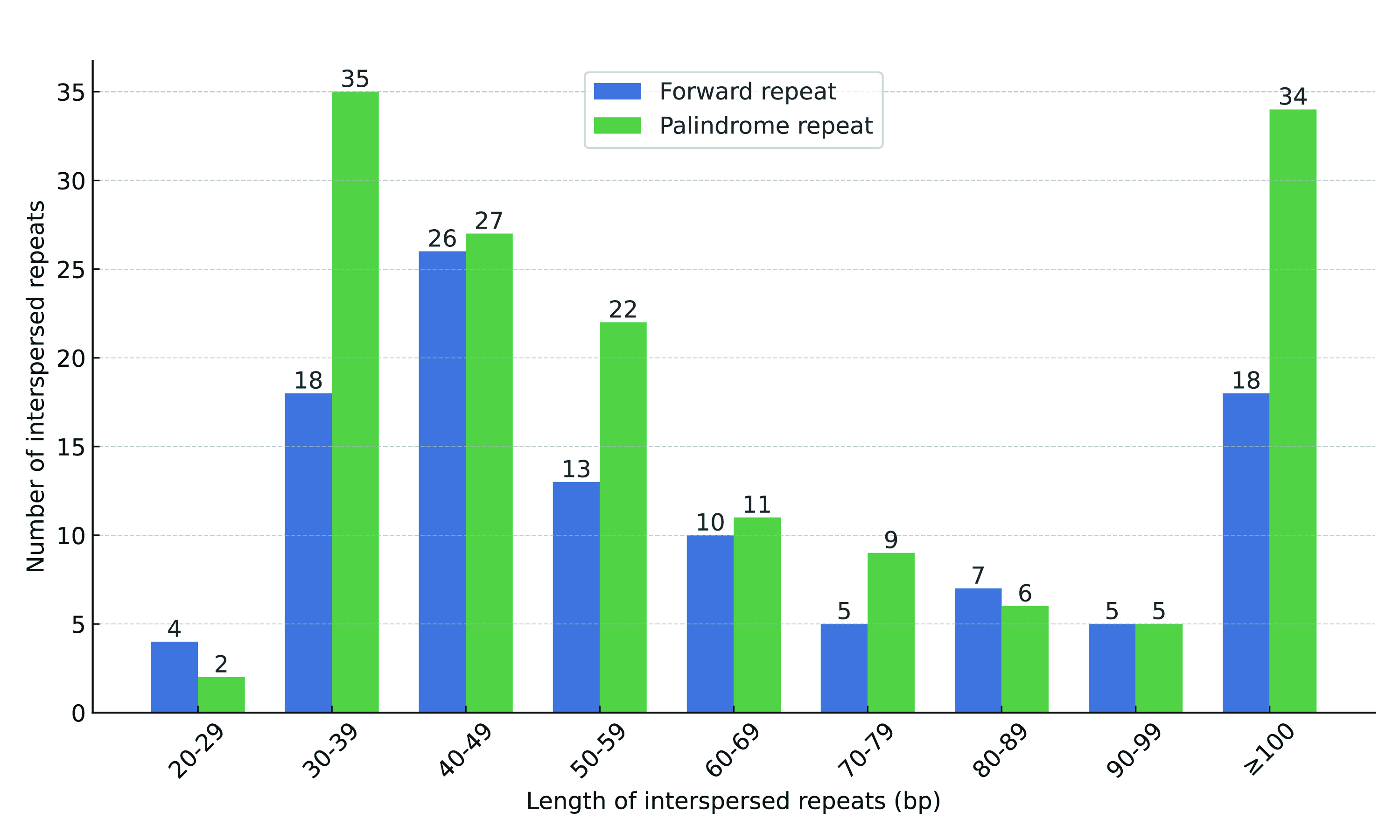


**Supplementary Figure 2.** Distribution of lengths of interspersed repeats in the *H. tibetana* mitogenome.


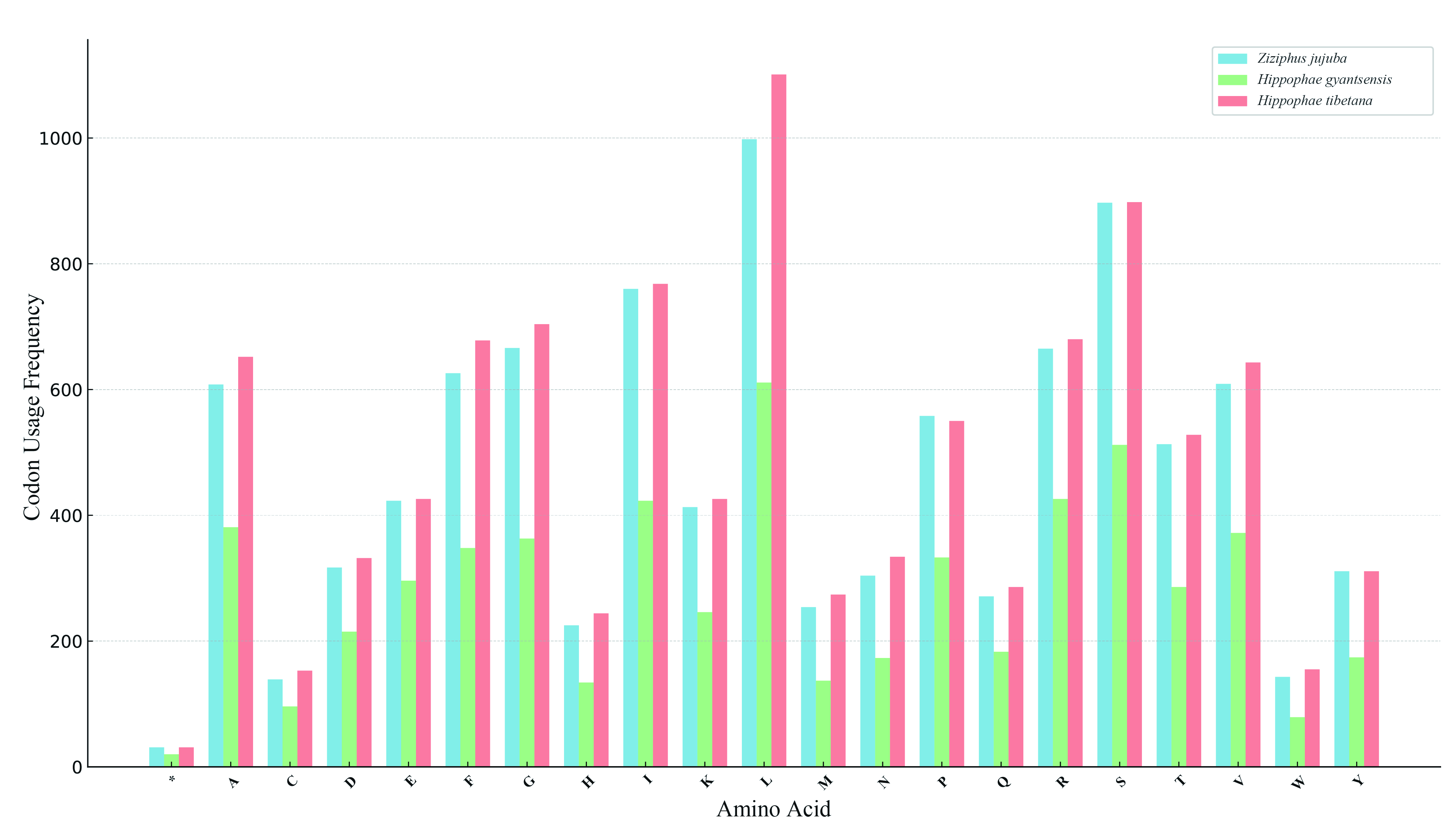


**Supplementary Figure 3.** Codon Usage Frequency by Amino Acid across Species. *: stands for stop codon；A: Ala, C: Cys, D: Asp, E: Glu, F: Phe, G: Gly, H: His, I: Ile, K: Lys, L: Leu, M: Met, N: Asn, P: Pro, Q: Gln, R: Arg, S: Ser, T: Thr, V: Val, W: Trp, and Y: Tyr.


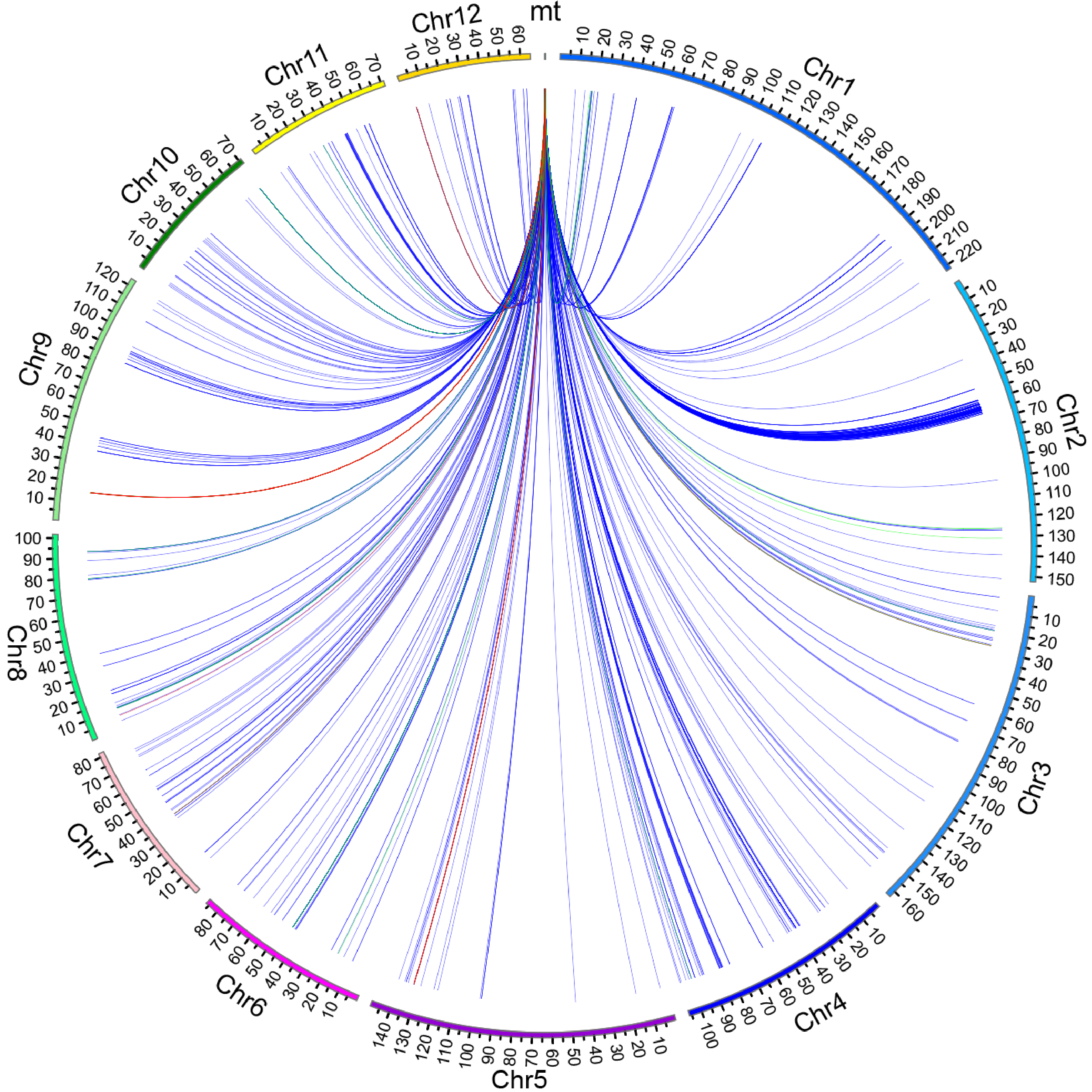


**Supplementary Figure 4.** Homologous fragments between the mitochondrial genome and the *H. tibetana* genome. In the figure, "mt" represents the mitochondrial genome, and the outermost labels represent the chromosome numbers of the *H. tibetana* genome. The outer ring's different colors represent different chromosomes and indicate the lengths of these chromosomes. Within the inner part of the circle, homologous sequences of different length ranges are indicated by different colors: blue for 1,000 - 5,000 bp, green for 5,000-10,000 bp, and red for >10,000 bp.


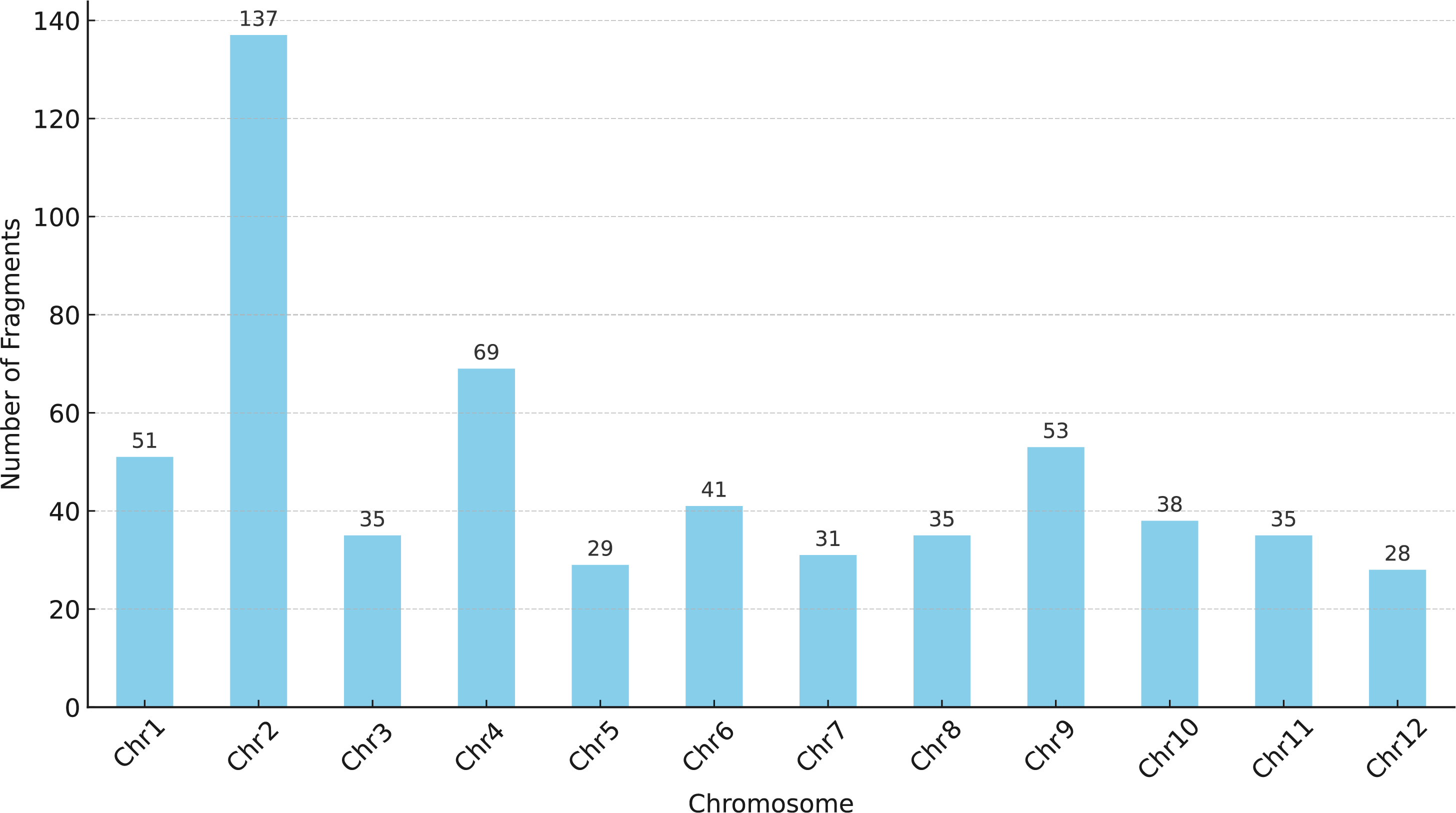


**Supplementary Figure 5.** The number of mitochondrial fragments transferred to the *H. tibetana* genome.


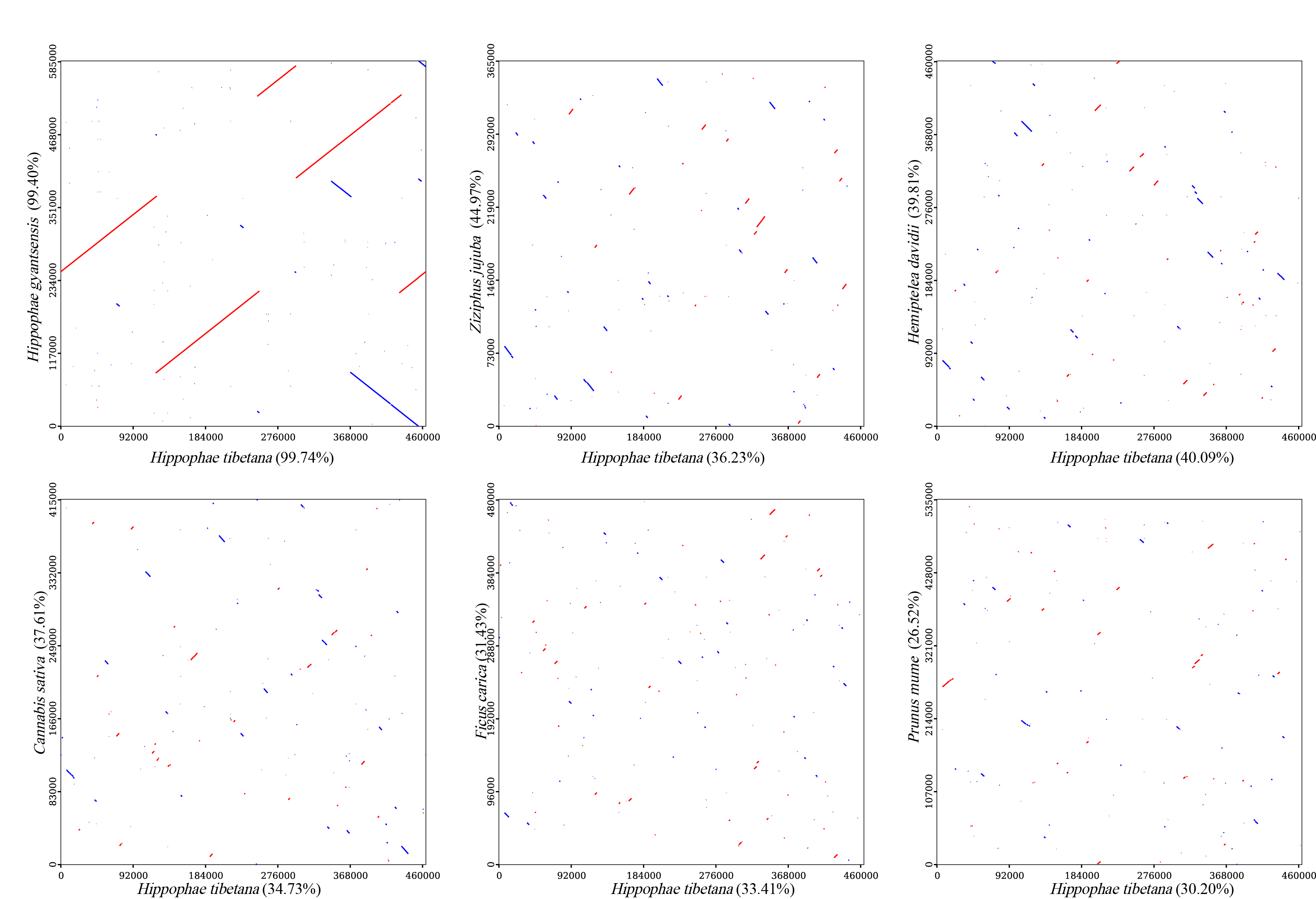


**Supplementary Figure 6.** Dot plot of *H. tibetana* with closely related species. The horizontal coordinate in each box indicates the assembled sequence, the vertical coordinate indicates the other sequences, the red line in the box indicates the forward comparison, and the blue line indicates the reverse complementary comparison.
